# Detecting surface changes in a familiar tune: exploring pitch, tempo and timbre

**DOI:** 10.1007/s10071-022-01604-w

**Published:** 2022-02-09

**Authors:** Paola Crespo-Bojorque, Alexandre Celma-Miralles, Juan M. Toro

**Affiliations:** 1 Universitat Pompeu Fabra, C. Ramon Trias Fargas, 25-27, 08005 Barcelona, Spain; 2 Royal Academy of Music in Aarhus/Aalborg, Aarhus University, Universitetsbyen, 3, 8000 Aarhus, Denmark; 3 Institució Catalana de Recerca I Estudis Avançats (ICREA), Pg. Lluís Companys, 23, 08019 Barcelona, Spain

**Keywords:** Music cognition, Pitch, Tempo, Timbre, Rats, Familiarization

## Abstract

Humans recognize a melody independently of whether it is played on a piano or a violin, faster or slower, or at higher or lower frequencies. Much of the way in which we engage with music relies in our ability to normalize across these surface changes. Despite the uniqueness of our music faculty, there is the possibility that key aspects in music processing emerge from general sensitivities already present in other species. Here we explore whether other animals react to surface changes in a tune. We familiarized the animals (Long–Evans rats) with the “Happy Birthday” tune on a piano. We then presented novel test items that included changes in pitch (higher and lower octave transpositions), tempo (double and half the speed) and timbre (violin and piccolo). While the rats responded differently to the familiar and the novel version of the tune when it was played on novel instruments, they did not respond differently to the original song and its novel versions that included octave transpositions and changes in tempo.

**Supplementary Information:**

The online version contains supplementary material available at 10.1007/s10071-022-01604-w.

## Introduction

Much like we can understand a given sentence independently of the gender or accent of the speaker, and even if it is shouted or whispered, the recognition of a familiar tune is done effortlessly regardless of the specific instrument it is used to play it or the speed and octave at which it is played. For instance, at every birthday party, the happy birthday song is sung by different people (with varying individual voices) at distinct frequency ranges and at a randomly-chosen speed (at a slower or faster tempo depending on the enthusiasm and general mood). Despite that, all the members of the party will identify the song, even those who know the lyrics in a different language. This is because we identify a musical excerpt as an object that can flexibly vary in at least these three dimensions (i.e. pitch, tempo and timbre) without losing its identity. In fact, this melody recognition ability is so pervasive in humans that is already present in infants as young as two-month olds (Plantinga and Trainor 2009), and is one of the building blocks upon which music appreciation is based. But to what extent does humans’ biological predisposition to process music emerge from sensitivities already present in non-human animals? In the present study we explore whether a distant non-vocal learner species, the rat (*Rattus norvegicus*), detects surface changes in a familiar tune.

The universality of key components in music has attracted much attention in recent years (Mehr et al. 2019). For example, there are open questions about whether certain common features present in music (e.g. reliance on simple frequency ratios, or variations along rhythmic and harmonic complexity) emerge from specific perceptual and cognitive constraints that predate the emergence of music. One way to address this issue has been by exploring the extent to which these features might arise from sensitivities that are already present in other animals (Fitch 2006; Hoeschele et al. 2015). Some earlier studies explored whether different animals can discriminate among musical styles using a variety of cues. For instance, pigeons (Porter and Neuringer 1984), rats (Okaichi and Okaichi 2001) and even carps (Chase 2001) have been shown to discriminate excerpts of songs drawn from different musical traditions (e.g. Bach versus Stravinsky, or blues versus classical music). However, since the contrasting excerpts varied along several dimensions, it is difficult to identify the specific features that the animals were using as a cue to guide their discrimination. Other studies identified more specific musical features that can be detected by other animals. For instance, both mammalian and avian species can perceive changes in fundamental frequency (i.e. musical pitch; e.g. rhesus monkeys [*Macaca mulatta*; Wright et al. 2000]), speed (i.e. musical tempo; e.g. California sea lion [*Zalophus californianus*; Cook et al. 2013]; cockatoo [*Cacatua galerita eleonora*; Patel et al. 2009]) and spectral envelope (i.e. musical timbre; e.g. chimpanzees [*Pan troglodytes*; Kojima and Kiritani 1989]; black-capped chickadees [*Poecile atricapillus*; Hoeschele et al. 2014]; zebra finches [*Taeniopygia guttata*; Ohms et al. 2010]). However, a key aspect in how humans process music is that we perceive musical structures in a relative instead of in an absolute way; that is, independently of surface changes along features such as pitch, tempo and timbre. It is thus important to understand the extent to which this ability is based on sensitivities already present in other species.

**Fig. 1:**
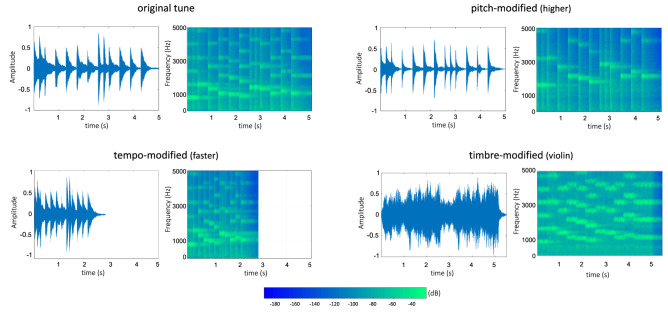
Amplitude waveforms and spectrograms of the original tune (played at 140 bpm on a piano with the tonic centered at C_6_; resolution of 1024 fast Fourier transform size and a Hamming window, 50% overlap), and one of its pitch-modified versions (the tonic centered at C_7_), one of its tempo-modified versions (played at 280 bpm) and one of its timbre-modified versions (played on a violin)

In the present study, we use the rat (*Rattus Norvegicus*) as a model to explore the detection of changes in pitch, tempo and timbre in a tune. Rats produce two types of ultrasonic vocalizations, aversive (at 22 kHz) and appetitive (at 50 kHz), by releasing air through the vocal tract (Brudzynski 2014). Importantly, rats can discriminate when the fundamental frequency and duration of these vocalizations change (Brudzynski 2014; Simola and Brudzynski 2018). But there is no evidence that such vocalizations are learned or that are composed by an organization of categorically different segments as are the vocalizations produced by songbirds and humans. Thus, findings with this species can be detached from their use of complex vocalizations and thus contribute to the understanding of the perceptual sensitivities underlying the origins of musicality. We familiarized the animals with an excerpt of the Happy Birthday song (the second half of the “*Happy Birthday”* song, composed by 13 tones, containing all the pitches of the Western major musical scale, while the tonic [C_6_] occupies a centric position in the frequency range of the sounds) played at the metronomic 140 bpm, using the timbre of an acoustic piano. After the familiarization, the animals were presented with three different test sessions. Each test session included two types of items: the familiar excerpt and a modified version of it (see Fig. 1). In the Pitch test, the octave of the melody was transposed (both higher [C_7_] and lower [C_5_]). In the Tempo test, the melody was played at different speeds (both faster [280 bpm] and slower [70 bpm]). In the Timbre test, the melody was played using the timbre of different instruments (violin and piccolo).

## Methods

### Subjects

Forty female Long–Evans rats *(Rattus norvegicus)* of 5 months of age were used in the study. Rats were housed in pairs and were exposed to a 12-h/12-h light–dark cycle. Animals had water ad libitum and were food-deprived, maintained at 85–90% of their free-feeding weights. Food was delivered after each familiarization session. Rats produce two types of ultrasonic vocalizations (at 22 and 50 kHz) by releasing air through the vocal tract, but they have not been shown to be vocal learners (Brudzynski 2013). Their hearing range is between the 200 Hz to 90 kHz (Fay 1988; Heffner et al. 1994; Warfield 1973). More importantly, they have been shown to readily detect variations for musical stimuli within the range of those used in the present study (Celma-Miralles and Toro 2020a, b; Crespo-Bojorque and Toro 2015, 2016).

### Apparatus

For the experiment, the rats were placed individually in Letica L830-C response boxes (Panlab S. L., Barcelona, Spain). Each box was equipped with an infrared detector located in the pellet feeder to register nose-poking responses. A custom-made software (RatBoxCBC) controlled the presentation of stimuli, recorded nose pokes, and delivered food. A Pioneer A-445 stereo amplifier and two Electro-Voice S-40 loudspeakers (with a response range from 85 Hz to 20 kHz), located beside the boxes, were used to present the stimuli at 81 dB SPL (as measured from the middle of the response box).

### Stimuli

**Fig. 2:**
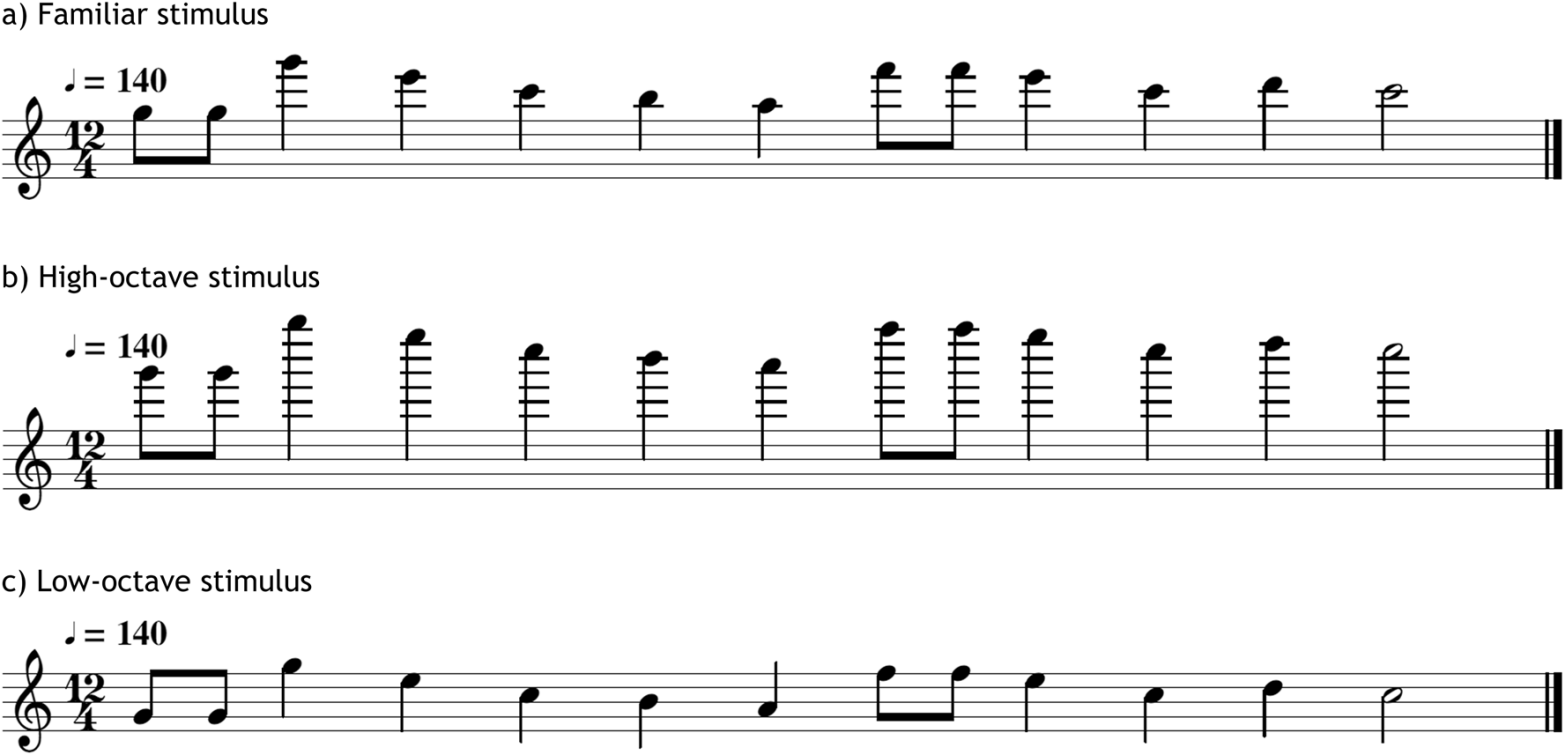
Music score for the familiar stimulus and its pitch-modified versions. One novel version is shifted one octave higher, while the other is shifted one octave lower

For the familiarization phase, the second half of the “*Happy Birthday”* song was used. The melody was composed by 13 tones, contained all the pitches of the Western major musical scale, and the tonic (C_6_) occupied a central position in the frequency range of the sounds. The tones were synthetized with MuseScore 2.2.2 (www.musescore.org) with the timbre of an acoustic piano. The tones included eight different pitches (see Fig. 2): G5 (783.9 Hz), A5 (880 Hz), B5 (987.8 Hz), C6 (1046.5 Hz), D6 (1174.7 Hz), E6 (1318.5 Hz), F6 (1396.9 Hz) and G6 (1568 Hz). Each sequence of 13 tones lasted 5,156 ms and contained three kinds of rhythmic figures. That is, the tones could have 3 different durations: 1 half note (857.14 ms), 8 quarter notes (428.5 ms) and 4 eight notes (214.28 ms). The tempo of the beat (every quarter note) occurred at the frequency of 2.33 Hz, at the metronomic 140 bpm.

For the test sessions, three different types of unfamiliar stimuli were created by modifying the familiar song along pitch, tempo and timbre (see Table 1). For the Pitch test, we created two novel stimuli (Higher and Lower) by changing the absolute frequency of the tones. For the high-pitch version of the test stimuli, we shifted the melody upwards one octave. The resulting tones were G6 (1568 Hz), A6 (1760 Hz), B6 (1975.5 Hz), C7 (2093 Hz), D7 (2349.3 Hz), E7 (2637 Hz), F7 (2793) and G7 (3135.9 Hz). For the low-pitch version of the test stimuli, we shifted the melody downwards one octave. The resulting pitches were G4 (391.9 Hz), A4 (440 Hz), B4 (493.9 Hz), C5 (523.3 Hz), D5 (587.3 Hz), E5 (659.3 Hz), F5 (698.5 Hz) and G5 (783.9 Hz). For the Tempo test, we created 2 novel stimuli (Faster, Slower) by changing the frequency of the beat. For the faster version of the test stimuli, the beat was speed up to 4.67 Hz (i.e., 280 bpm; the double of the original tempo). For the slower version of the test stimuli, the beat was slowed down to 1.17 Hz (i.e., 70 bpm; half of the original tempo). Finally, for the Timbre test, we created 2 novel stimuli (Violin, Piccolo) by changing the instrument playing the original tune. Two instrumental sounds belonging to new families of instruments were used: violin (string family) and piccolo flute (woodwind family). All the melodies presented to the animals fall well within the hearing range of the rats (Fay 1988; Warfield 1973), can be readily discriminated at the intensity that the stimuli were presented (Heffner et al. 1994), and stimuli with similar characteristics have successfully been used in previous studies exploring the detection of acoustic changes in rats (Celma-Miralles and Toro 2020a, b; Crespo-Bojorque and Toro 2015; D’Amato and Salmon 1982; Poli and Previde 1991).

**Table 1:** Stimuli parameters used during familiarization and tests

	Familiarization	Test	
Pitch		Higher	Lower
	C_6_ (1046.5 Hz)	C_7_ (2093 Hz)	C_5_ (523.3 Hz)
Tempo		Faster	Slower
	140 bpm	280 bpm	70 bpm
Timbre	Keyboard Piano	String Violin	Woodwind Piccolo

### Procedure

The experiment consisted of a familiarization phase followed by three test sessions. During familiarization, 20 sessions were run, one 10-min session per day. In each session, the rats were placed individually in a response box and were presented with 40 repetitions of the familiarization melody. The melody was played with an inter stimuli interval (ITI) of 8 s. During the ITI, the animals were presented with a 45 mg-sucrose food pellet after nose-poke responses using a variable ratio of 5 ± 2 (so, every time the animal responded between three and seven times a pellet would be delivered). In this sense, the present procedure differs from a classical go/no-go paradigm, as the rat is not punished for producing responses after non-target stimuli. In fact, during the familiarization phase, only target stimuli were presented. After the familiarization phase, three test sessions were run (Pitch test, Tempo test and Timbre test). There was one familiarization session before each test session. The animals were divided into three groups so that the order of the presentation of the three tests was balanced across groups. The test sessions were similar to the familiarization sessions. The only difference was that 20 test stimuli (10 modified versions and 10 original items) replaced 20 familiarization items. The order of presentation of the stimuli was randomized within each test session, with the only constrain that no more than 2 items of the same type were presented in a row. No pellets were delivered after the presentation of test stimuli independently of the animals’ responses. All the experimental procedures were conducted in accordance with the Catalan, Spanish and European guidelines and regulations for the treatment of experimental animals and received the necessary approval from the ethics committee of the Universitat Pompeu Fabra and the Generalitat de Catalunya (protocol number 10557).

## Results

**Fig. 3:**
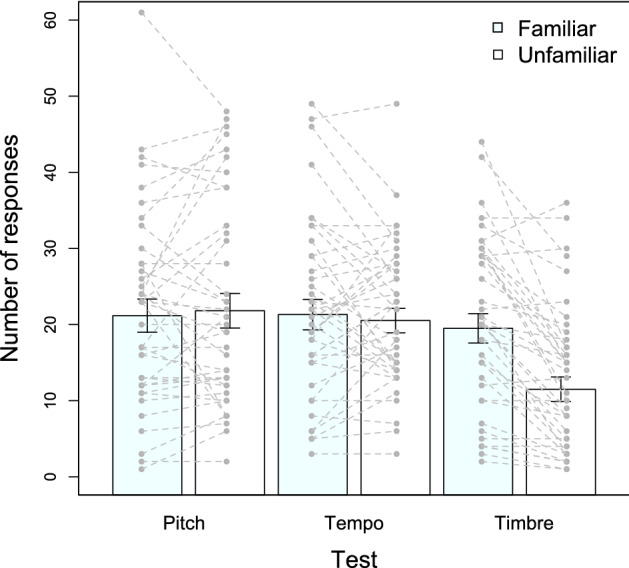
Individual number of responses (single dots) to the familiar (original tune; light blue) and unfamiliar (white) test stimuli across the three tests. Bars show mean and standard error at the group level. Dotted lines show individual changes within each test

We compared the mean number of responses to the *familiar* test stimuli (original melody) to the mean number of responses to the *unfamiliar* test stimuli (modified versions) across the three tests. A Repeated-Measures ANOVA with the within factors Familiarity (familiar, unfamiliar) and Test (Pitch, Tempo, Timbre) revealed an effect of Familiarity (*F*_(1,39)_ = 11.325, *p* = 0.002, *η*^2^ = 0.225) and Test (*F*_(1.57,61.11)_ = 3.604, *p* = 0.044, *η*^2^ = 0.085), as well as an interaction between them (*F*_(2,78)_ = 12.155, *p* < 0.001, *η*^2^ = 0.238). The Greenhouse–Geisser correction for violations of sphericity was used. Post-hoc pairwise comparisons with the Bonferroni alpha correction revealed that the animals’ responses did not differ between the familiar melody (*M* = 21.83, SD = 12.70) and the versions with transposed octaves (Pitch test; *M* = 22.43, SD = 13.35; MD =  − 0.60, *p* = 0.640); nor between the familiar melody (*M* = 21.95, SD = 11.71) and the versions with novel tempi (Tempo test; *M* = 21.23, SD = 9.38; MD = 0.73, *p* = 0.584). In contrast, the animals produced more responses after the familiar melody (*M* = 20.28, SD = 11.25) than after the versions played with new instruments (Timbre test; *M* = 12.88, SD = 9.41; MD = 7.40, *p* < 0.001; see Fig. 3). There were no significant differences between the responses to familiar test stimuli across test sessions (all *p* = 1), but the responses to unfamiliar test stimuli were smaller for the Timbre test compared to the Pitch (MD = − 9.55, *p* = 0.007) and to the Tempo (MD = − 8.35, *p* = 0.001) tests. To explore if there were any changes across sessions for each test, we conducted separate repeated-measures ANOVA for the Pitch, the Tempo and the Timbre tests. For the Pitch Test, we observed a significant effect of Session (*F*_(2, 37)_ = 19.91, *p* < 0.001, *η*^2^ = 0.469) on the number of responses to test stimuli but no significant effects of Familiarity (*F*_(1, 37)_ = 0.249, *p* = 0.621, *η*^2^ = 6.228e−4) or the interaction between them (*F*_(2,37)_ = 0.52, *p* = 0.519, *η*^2^ = 0.003). Similarly, for the Tempo test, results show a significant effect of Session (*F*_(2, 37)_ = 3.487, *p* = 0.041, *η*^2^ = 0.134), but no significant effects of Familiarity (*F*_(1, 37)_ = 0.267, *p* = 0.609, *η*^2^ = 0.001) or interaction between them (*F*_(2,37)_ = 0.257, *p* = 0.775, *η*^2^ = 0.002). Thus, rats produced more nose-poking responses from session 1 to session 3, but crucially, this increase in responses did not have any effect in the pattern of responses that they were producing after familiar and unfamiliar stimuli. In contrast, for the Timbre test, results show a significant effect of Familiarity (*F*_(1, 37)_ = 40.54, *p* =  < 0.001, *η*^2^ = 0.116), as the animals consistently responded more to the familiar than to the unfamiliar stimuli, but no significant effects of Session (*F*_(2, 37)_ = 2.862, *p* = 0.070, *η*^2^ = 0.104) or interaction between them (*F*_(2,37)_ = 0.812, *p* = 0.452, *η*^2^ = 0.005). We also explored if there were any effects from the order in which the tests were presented. We observed an effect of Order (*F*_(2, 78)_ = 25.943, *p* < 0.001, *η*^2^ = 0.305), as the animals presented with the Pitch test first produced more responses than the animals in the other groups. We also observed an effect of Familiarity (*F*_(1, 39)_ = 11.941, *p* = 0.001, *η*^2^ = 0.019), but crucially, the interaction between factors was not significant (*F*_(2, 78)_ = 0.596, *p* = 0.553, *η*^2^ = 0.002).

To further explore if the lack of significant differences in responses that we observed in the Pitch and Tempo tests was a failure of the animals to discriminate between test items, or rather a true null result in which the data is actually providing information about the null hypothesis, we ran Bayesian paired sample *t* tests (JASP Team, 2020). The data, in fact, provide no support for the alternative hypothesis neither for the Pitch test (BF_10_ = 0.189), nor for the Tempo test (BF_10_ = 0.197), but it does provide moderate support for the null hypothesis for both of them (Pitch [BF_01_ = 5.279, error % 9.153e−6]; and Tempo [BF_01_ = 5.081, error % 8.807e−6]). This suggests that the animals are generalizing their responses from the familiar stimuli to the novel ones. So, it is not that the animals are behaving randomly, but rather, they are responding to the novel test stimuli as if it is functionally equivalent to the familiar stimuli. For the Timbre test, a Bayesian paired sample t-test confirm strong evidence in favor of the alternative hypothesis (BF_10_ = 95,059.227, error % 2.729e−10).

**Fig. 4:**
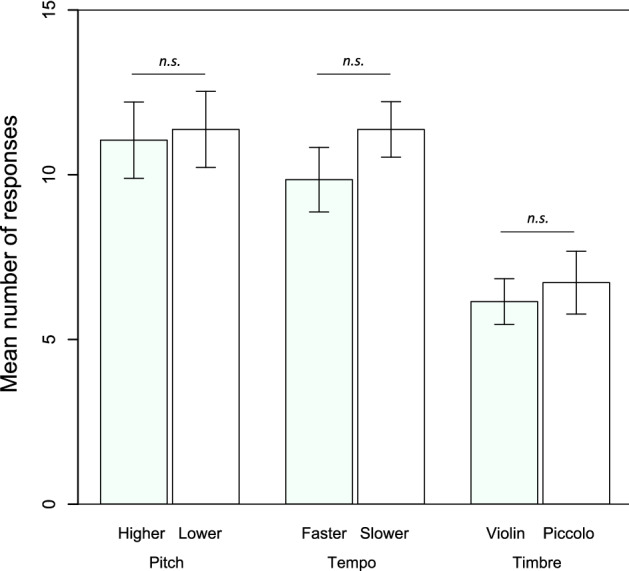
Mean number of responses and standard error bars to the two different types of unfamiliar stimuli across the three tests

We also explored any possible bias towards high or low frequencies, fast or slow tempo, or the timbre of the instruments by comparing the responses to the two kinds of unfamiliar stimuli we used during each test. We did not find differences between the higher frequency (*M* = 11.05, SD = 7.32) and the lower-frequency (*M* = 11.38, SD = 7.31) test items (*t*(39) − 0.34, *p* = 0.733, 95% CI [− 2.24, 1.59]) in the Pitch test; between the faster tempo (*M* = 9.85, SD = 6.19) and the slower tempo (*M* = 11.38, SD = 5.32) test items (*t*(39) − 1.44, *p* = 0.159, 95% CI [− 3.67, 0.62]) in the Tempo test; nor between the violin (*M* = 6.15, SD = 4.37) and the piccolo (*M* = 6.73, SD = 6.03) test items (*t*(39) − 0.77, *p* = 0.447, 95% CI [− 2.10, 0.94]) in the Timbre test (see Fig. 4; individual data are reported in the supplementary material accompanying this article).

## Discussion

Research using a variety of species and techniques has shown that there are universal principles in how features such as pitch, intensity and duration are modulated during the production of complex vocalizations in birds and humans (Mann et al. 2021; Tierney et al. 2011). Across different species, vocalizations tend to have an arc-shaped pitch contour and have longer segments at the end, likely linked to motor constraints on how these vocalizations are made (Tierney et al. 2011). Such principles seem to constrain how sequences varying in pitch and duration are grouped in humans (Iversen et al. 2008) and animals (Spierings et al. 2017; Toro and Crespo-Bojorque 2021) by following what is known as the Iambic–Trochaic law. Similarly, some common aspects in how humans process music might emerge from general sensitivities that are already present in other species (Fitch 2006; Patel 2019). In this study, we explored whether other animals react to modifications in fundamental frequency, speed and spectral envelope over a familiar tune. Our results show that rats generalize their learned responses over melodies shifted an octave upward or downward, and over melodies accelerated or deaccelerated to double or half tempi. On the contrary, the rats produce less responses when they are presented with melodies in which we changed the piano timbre into a violin or a piccolo timbre.

## Pitch

Pitch is a quality defined by the rate of vibrations producing a sound (with a fundamental frequency and overtones) and in music it is perceived in the context of other tones. In humans, tones separated by an octave (i.e. doubling in frequency) are perceived as similar (Hoeschele et al. 2012; Patel 2008). This is known as octave equivalence. The octave is inherent in vocal productions (natural vocalizations) and humans show octave generalization when they sing or speak (Hoeschele et al. 2012). In the present study, we observed evidence suggesting that the rats generalize across changes in the fundamental frequencies of the tones composing the melody. When the frequency of the melody was shifted one octave up or down, the animals did not respond differently to the familiar and the novel tune, suggesting that the rats perceived as equivalent the melodies irrespective of their changes in octaves.

An alternative explanation of these results is that the rats’ failure to respond differently to the familiar song and its octave-transposed versions might be the result of the rats not being able to actually perceive physical changes in the range that we implemented them. That is, that rats cannot perceive changes in the fundamental frequency of sounds between 1046.5 Hz (familiar song) and 2093 Hz or 523.3 Hz (modified versions) when presented at 81 dB. This is not the case. Rats can readily discriminate between sounds changing in fundamental frequency in the ranges used in the present study (Astikainen et al. 2014; de la Mora et al. 2013; Eriksson and Villa 2006; Nakamura et al. 2011). Moreover, these fundamental frequencies are readily discriminated at the intensity that we presented them (Heffner et al. 1994). Our results also parallel those reported with Albino rats (d’Amato and Salmon 1982). In their experiment, the animals learned to discriminate between two tunes, and generalized their discrimination when one of the tunes was raised one octave. Similar generalization across octaves has been observed in other mammals, such as rhesus monkeys (Wright et al. 2000). However, these results seem to contrast with reported difficulties in songbirds to transfer responses across different octaves. For example, no evidence has been found that chickadees can transfer tone discriminations across octaves (Hoeschele et al. 2013), and similar results have been observed with budgerigars (Wagner et al. 2019) and European starlings (Cynx 1993). Interestingly, neither rats nor rhesus monkeys are vocal learners, while chickadees, budgerigars and starlings are. It is thus possible that the ability to learn complex vocalizations might interact with the generalization across octaves. Complementarily, octave equivalence in rats (for example D’Amato and Salmon 1982; present experiment) and monkeys (Wright et al. 2000) has been observed with the use of relatively complex tunes, while the experiments with the songbirds relied on the presentation of individual tones. It might thus be the case that instances of octave equivalence are linked to the possibility to track pitch intervals (that remain constant over changes in key) that define harmonic structure instead of the specific frequencies at which the musical notes are played.

## Tempo

The second dimension that we manipulated in the present study was the speed at which the song was presented, commonly referred to as tempo. When we changed the speed of the song to slower and faster tempi, we did not observe significant changes in the animals’ responses. A possible interpretation of these results is that the rats learned to identify the temporal relations between the tone durations defining the familiar tune independently of the speed at which they were presented. As in the pitch condition, it might also be the case that the similar responses that the rats produced to the familiar and the tempo-modified versions of the song were the result of the animals not being able to discriminate between sequences of tones presented at 140 bpm (familiar song) from sequences of tones presented at 280 bpm or 70 bpm. Similarly, that rats might not perceive changes in duration between 5,156 ms (familiar song) and 2,571 ms or 10,285 ms (modified versions). But several experiments have demonstrated that rats can perceive changes in the speed of presentation of sequences of sounds (Katsu et al. 2021) and that they readily discriminate between changes in duration similar to the ones we used here (Nakamura et al. 2011; Roger 2009; Toro and Nespor 2015). Further evidence suggests that, if trained, rats can use temporal regularities to discriminate among sequences of tones independently of distinct tempi (Celma-Miralles and Toro 2020a). That is, they can learn to discriminate between sequences composed by regular from sequences composed by irregular intervals, independently of their absolute durations. Even more, the animals can detect changes in the temporal relations between tones of a song (Celma-Miralles and Toro 2020b). The present results provide further evidence suggesting that, when these relations are maintained, and only the rate of presentation (the tempo) changes, the animals do not seem to react to that change. This suggests that the animals can focus on the temporal aspects of the tune and generalize across what might be considered as surface changes, such as speed of presentation.

The fact that the rats are not responding to changes in fundamental frequency and speed over a sequence of tones may be related to the processing of intra-species vocal communication signals (Brudzynski 2014; Simola and Brudzynski 2018). Rats produce distinct ultrasonic vocalizations for positive and negative states (Saito et al. 2016), and similar to humans, rats show sexual dimorphism in their vocalizations (Lenell and Johnson 2017 [this is also present in mice, Warren et al. 2018]). Male rats produce ultrasonic vocalizations that are lower in pitch compared to those of female rats. This means that they may need to decode and recognize the vocalizations by focusing on general relations among tones in a particular frequency range, rather than on the absolute pitch or mean frequency. In addition, to recognize and appropriately react to similar kinds of vocalizations, rats must be able to flexibly process faster and slower versions of ultrasonic vocalizations, identify general durational features and adapt to the specific underlying timings of the individual producing them (Brudzynski 2014). These normalization processes used for inter-specific vocalizations might be at the root of the pattern of responses that we observed in the present study.

## Timbre

Contrary to what we observed when we changed the frequency or the tempo of the familiar tune, when we changed the instrument used to play it (from a piano to a violin or a piccolo), the rats responded as if it was a different melody. Timbre is the acoustic property differentiating the same note (a tone with identical pitch, intensity and duration), played in a piano or a violin. Timbre is defined by changes in the spectral envelope of a sound: how energy is distributed across different frequencies. Previous research using a small sample of Long–Evans rats suggested that they can use changes in instrument as a discrimination cue (Poli and Previde 1991). In the experiment, the animals learned to respond differently to two versions of the same tune, one played by guitar and another played by trumpet. Experiments with songbirds have also shown that they readily detect similar changes in timbre. For example, zebra finches and budgerigars can detect relatively small changes in the amplitude of key harmonics that correlate with changes in timbre (Lohr and Dooling 1998), while Black-capped chickadees (Hoeschele et al. 2014) and European starlings fail to recognize chords and tone sequences when their timbre changes (experiment 2 in Bregman et al. 2016). Similarly, Long Evans rats also fail to discriminate between languages if the person producing the sentences changes (Toro et al. 2005). In the present study, when we changed the instrument playing the familiar tune, the rats responded as if it was a different tune. Changes in timbre arise from changes in amplitudes across the frequency spectrum. The converging results observed across different species seem to confirm that such changes are readily used for animals to identify sequences of sounds. In fact, spectral cues have been shown to be pivotal for the recognitions of patterns in songbirds (Bregman et al. 2016). The fact that a sensitivity for changes in amplitude across the frequency spectrum has been observed across avian and mammal species, and across vocal and non-vocal learning species, suggests that it is not directly linked to the ability to produce complex vocalizations.

The rats’ reaction to changes in timbre parallels the results observed with Black-capped chickadees (Hoeschele et al. 2014) and European starlings (Bregman et al. 2016), but it contrasts with the observation that humans readily recognize tunes across different timbres. One possibility is that such difference might emerge from humans’ need to normalize the speech signal across different speakers to effectively communicate through language (Pisoni and Remez 2005). That is, humans readily process linguistic information independently of the identity of the speaker. This normalization mechanism used for language processing might have been co-opted in the music domain to allow for the recognition of melodies independently of the instrument being used to play them (Hoeschele et al. 2014). Lacking such normalization mechanism, the animals in our study might find it difficult to recognize a melody when different instruments produce it.

Could it be that in our study the rats are only perceiving the instrument used to play the tune and cannot even discriminate between excerpts that are played on the same instrument? Several experiments suggest that this is not the case. Studies with rats have shown that they are able to discriminate between sequences of syllables produced by the same speaker (Toro et al. 2005) and between sequences of tones played by the same instrument (Celma-Miralles and Toro 2020a, b; Crespo-Bojorque and Toro 2015; de la Mora et al. 2013) Similarly, a wide array of species can identify rhythmic (Ravignani et al. 2019) and harmonic (Patel 2019) patterns in music in the absence of changes in timbre. Timbre thus seems to be a salient feature for melody recognition in non-human animals. But in the absence of timbre changes, several species (including rats) are able to track the temporal and harmonic relations among tones that define a tune. Our results, however, open the door to further studies exploring possible differences across species regarding the processing of changes in the spectral envelope and its role in sequence recognition.

The contrast in patterns of responses that we observed when we changed the familiar tune’s frequency, speed and timbre suggests that these different features are not perceived and processed the same by rats. Even though there is convincing evidence that these animals can discriminate acoustic stimuli that changes in frequency and speed, they responded similar to the familiar and the novel pitch and tempo excerpts. The animals are thus generalizing their learned response from the familiar Happy Birthday excerpt to the novel ones, suggesting they have built an equivalence class along stimuli presented at different frequencies and speeds (for a discussion on generalization, see Cheng, 2002). A key question in our study is why the rats took variations in tempo and pitch to be less psychologically distant than changes in timbre. As we discussed above, this might be related to the processing of intra-species vocal communication signals. Because rats’ ultrasonic vocalizations contain changes in frequency and speed, the animals need to decode them by focusing on general frequency and durational relations among tones. In contrast, there is no indication that their vocalizations provide any relevant information over the temporal spectrum over which they need to normalize.

## Conclusion

A key aspect of studying humans’ biological predisposition to music is the extent to which the ability to identify constant relations between tones that define a song emerges from sensitivities already present in non-human animals. The present study explores rats’ sensitivities to changes along three dimensions, namely, fundamental frequency (pitch), speed (tempo) and spectral envelope (timbre). Our results show that rats respond similarly to a melody even if it is presented at different frequencies and speeds. The results also demonstrate that timbre (the frequency spectrum) provides a strong cue for the identification of sounds in non-human animals. Thus, the ability to normalize across surface musical features that is present in humans might partly emerge from pre-existing sensitivities to track harmonic and temporal patterns that are already present in other species. More research is, however, needed to understand how the ability to generalize over changes in the frequency spectrum emerged in the human lineage.

## Supplementary Information

Below is the link to the electronic supplementary material.

Supplementary file1 (DOCX 32 kb)

Supplementary file2 (WAV 889 kb)

Supplementary file3 (WAV 868 kb)

Supplementary file4 (WAV 962 kb)

Supplementary file5 (WAV 248 kb)

Supplementary file6 (WAV 2205 kb)

Supplementary file7 (WAV 1203 kb)

Supplementary file8 (WAV 1191 kb)

## Data Availability

All primary data are included in the Supplementary material.
