# Supplementary material for: Detecting surface changes in a familiar tune: exploring pitch, tempo and timbre
Source: Anim Cogn. 2022 Feb 9;25(4):951–60. doi: 10.1007/s10071-022-01604-w (PMC9334415; doi:10.1007/s10071-022-01604-w)
Supplement: Supplementary file 1 — Supplementary file1 (DOCX 32 kb) [file 10071_2022_1604_MOESM1_ESM.docx]

**Supplementary material**

**Detecting surface changes in a familiar tune: Exploring pitch, tempo and timbre**

Paola Crespo-Bojorque, Alexandre Celma-Miralles, Juan M. Toro

**S1 Primary data**

|  | **Pitch** | | **Tempo** | | **Timbre** | |
| --- | --- | --- | --- | --- | --- | --- |
| **Rat** | **Familiar** | **Unfamiliar** | **Familiar** | **Unfamiliar** | **Familiar** | **Unfamiliar** |
|  |  | (higher-lower) |  | (faster-slower) |  | (violin-piccolo) |
| **1** | 1 | 6 | 19 | 28 | 4 | 2 |
| **2** | 3 | 8 | 6 | 13 | 5 | 5 |
| **3** | 20 | 20 | 29 | 15 | 12 | 13 |
| **4** | 27 | 23 | 41 | 26 | 10 | 8 |
| **5** | 17 | 12 | 6 | 18 | 18 | 6 |
| **6** | 23 | 8 | 19 | 29 | 16 | 5 |
| **7** | 23 | 19 | 18 | 14 | 30 | 11 |
| **8** | 10 | 11 | 8 | 7 | 21 | 15 |
| **9** | 11 | 10 | 10 | 10 | 18 | 16 |
| **10** | 12 | 14 | 15 | 19 | 3 | 1 |
| **11** | 13 | 13 | 34 | 24 | 13 | 3 |
| **12** | 6 | 7 | 16 | 23 | 30 | 16 |
| **13** | 17 | 17 | 24 | 15 | 15 | 4 |
| **14** | 2 | 2 | 3 | 3 | 42 | 29 |
| **15** | 11 | 13 | 16 | 16 | 22 | 23 |
| **16** | 12 | 21 | 5 | 11 | 28 | 15 |
| **17** | 16 | 19 | 16 | 23 | 31 | 17 |
| **18** | 33 | 24 | 5 | 6 | 6 | 2 |
| **19** | 12 | 13 | 25 | 18 | 4 | 4 |
| **20** | 23 | 32 | 15 | 13 | 20 | 9 |
| **21** | 28 | 8 | 20 | 13 | 20 | 14 |
| **22** | 28 | 28 | 26 | 28 | 15 | 8 |
| **23** | 16 | 14 | 22 | 24 | 29 | 15 |
| **24** | 8 | 10 | 49 | 37 | 44 | 17 |
| **25** | 13 | 12 | 12 | 17 | 20 | 8 |
| **26** | 34 | 45 | 46 | 31 | 23 | 20 |
| **27** | 26 | 33 | 22 | 14 | 4 | 2 |
| **28** | 38 | 43 | 10 | 23 | 29 | 30 |
| **29** | 24 | 21 | 29 | 32 | 21 | 3 |
| **30** | 61 | 48 | 27 | 30 | 30 | 10 |
| **31** | 43 | 46 | 22 | 28 | 2 | 1 |
| **32** | 30 | 23 | 23 | 22 | 34 | 34 |
| **33** | 17 | 31 | 34 | 27 | 10 | 10 |
| **34** | 36 | 38 | 21 | 26 | 31 | 36 |
| **35** | 25 | 42 | 31 | 14 | 20 | 18 |
| **36** | 24 | 16 | 18 | 33 | 33 | 27 |
| **37** | 23 | 22 | 33 | 22 | 26 | 19 |
| **38** | 24 | 47 | 23 | 15 | 7 | 3 |
| **39** | 41 | 40 | 47 | 49 | 29 | 21 |
| **40** | 42 | 38 | 33 | 33 | 36 | 15 |

Total number of responses to Familiar and Unfamiliar test items across the three tests for each animal.

**S2**

|  | **Pitch** | | **Tempo** | | **Timbre** | |
| --- | --- | --- | --- | --- | --- | --- |
| **Rat** | **Higher** | **Lower** | **Faster** | **Slower** | **Violin** | **Piccolo** |
| **1** | 4 | 2 | 12 | 16 | 2 | 0 |
| **2** | 3 | 5 | 3 | 10 | 0 | 5 |
| **3** | 13 | 7 | 10 | 5 | 5 | 8 |
| **4** | 7 | 16 | 7 | 19 | 4 | 4 |
| **5** | 8 | 4 | 8 | 10 | 5 | 1 |
| **6** | 4 | 4 | 17 | 12 | 3 | 2 |
| **7** | 7 | 12 | 8 | 6 | 8 | 3 |
| **8** | 3 | 8 | 7 | 0 | 10 | 5 |
| **9** | 5 | 5 | 4 | 6 | 4 | 12 |
| **10** | 5 | 9 | 3 | 16 | 1 | 0 |
| **11** | 8 | 5 | 10 | 14 | 0 | 3 |
| **12** | 3 | 4 | 9 | 14 | 6 | 10 |
| **13** | 11 | 6 | 6 | 9 | 4 | 0 |
| **14** | 0 | 2 | 0 | 3 | 8 | 21 |
| **15** | 5 | 8 | 7 | 9 | 5 | 18 |
| **16** | 4 | 17 | 5 | 6 | 8 | 7 |
| **17** | 6 | 13 | 8 | 15 | 9 | 8 |
| **18** | 13 | 11 | 6 | 0 | 1 | 1 |
| **19** | 8 | 5 | 10 | 8 | 3 | 1 |
| **20** | 17 | 15 | 2 | 11 | 6 | 3 |
| **21** | 6 | 2 | 4 | 9 | 8 | 6 |
| **22** | 19 | 9 | 11 | 17 | 4 | 4 |
| **23** | 6 | 8 | 13 | 11 | 9 | 6 |
| **24** | 4 | 6 | 17 | 20 | 11 | 6 |
| **25** | 7 | 5 | 6 | 11 | 4 | 4 |
| **26** | 29 | 16 | 17 | 14 | 11 | 9 |
| **27** | 11 | 22 | 4 | 10 | 2 | 0 |
| **28** | 20 | 23 | 7 | 16 | 13 | 17 |
| **29** | 11 | 10 | 13 | 19 | 0 | 3 |
| **30** | 22 | 26 | 12 | 18 | 3 | 7 |
| **31** | 27 | 19 | 15 | 13 | 0 | 1 |
| **32** | 12 | 11 | 16 | 6 | 14 | 20 |
| **33** | 11 | 20 | 18 | 9 | 5 | 5 |
| **34** | 20 | 18 | 4 | 22 | 17 | 19 |
| **35** | 15 | 27 | 8 | 6 | 5 | 13 |
| **36** | 11 | 5 | 17 | 16 | 12 | 15 |
| **37** | 13 | 9 | 12 | 10 | 14 | 5 |
| **38** | 20 | 27 | 5 | 10 | 3 | 0 |
| **39** | 24 | 16 | 31 | 18 | 9 | 12 |
| **40** | 20 | 18 | 22 | 11 | 10 | 5 |

Total number of responses to unfamiliar test items across the three tests for each animal.
